# Factors affecting food waste: A bibliometric review on the household behaviors

**DOI:** 10.1371/journal.pone.0289323

**Published:** 2023-07-28

**Authors:** Vittoria Pilone, Naomi di Santo, Roberta Sisto

**Affiliations:** Department of Economics, Management and Territory, University of Foggia, Foggia, Italy; West Pomeranian University of Technology, POLAND

## Abstract

Sustainability issues such as food insecurity, climate change, land degradation, economic development and food waste are the actual most important challenges at the global level. Among them, the food waste (FW) challenge has a great magnitude, emphasizing the importance of examining this issue. Specifically, there is a need to focus on the household level. Thus, this study aims to investigate and identify the main factors influencing FW household behaviors on which policymakers and stakeholders could outline specific and sustainable strategies. Starting from a large number of published studies on this subject with a similar aim but focusing on specific Countries or contexts, the goal of our study is achieved through the implementation of a systematic literature review followed by a bibliometric review using the VOSviewer software. The selected query generated a total of 235 matching papers from which only 111 papers were collected for the bibliometric review because of the inclusion criteria. The analysis showed the existence of four major research strands: the largest one analyses the antecedents of behavior during food management, including the implementation of the Theory of Planned Behavior (TPB). Other detected topics are the economic impact of FW, the effects generated by the Covid-19 pandemic on consumer behaviors, and finally, the environmental and social effects of FW. The objective of this study is to investigate and identify the main factors influencing FW household behaviors. The obtained output represents useful information for policymakers and stakeholders to outline specific and sustainable strategies to reduce FW.

## 1. Introduction

Food Waste (FW) reduction, for its economic, social and environmental impacts represents a relevant issue either for the international scientific research or the political and social agenda in developed and modern societies [1–4]. Indeed, together with the growing relevance of the food-related issues in the collective consciousness [5], it is considered an emergency and a priority not only for local and national governments, but also for the European Union and the United Nations that, starting from 2015, included FW reduction among the 17 Sustainable Development Goals (SDGs).

More specifically, among them, the main challenges are related to Goal 2 “Zero Hunger” and Goal 12 “Responsible Consumption and Production”, with reference to Target 12.3, which requires, by 2030, to “halve per capita global food waste at the retail and consumer levels and reduce Food Loss (FL) along production and supply chains, including post-harvest losses”. To this aim, to accelerate the achievement of the 17 SDGs, the European Commission has published the “Closing the loop—An EU action plan for the Circular Economy” that contains a monitoring framework for the circular economy [6].

The great attention reserved to FW depends on its double bound to natural resources because, like food production, it depends on natural resources endowment and creates pressures on the environment. More specifically, it produces negative impacts on the landscape and ecosystem services, contributes extensively to biodiversity and water loss, greenhouse gas emissions and land degradation [7–9].

These consideration**s**, make FW a hot topic at global level, strictly linked to other global key challenges such as food security and malnourishment [10–12], climate change, and sustainable economic development [3].

Notwithstanding its global and concordant relevance, it important to highlight that, from a nomenclature point of view, there is still confusion between the two different expressions of "food waste" and "food loss", mainly by media and policy makers who in many cases use them indiscriminately. For this reason, the Swedish Institute for Food and Biotechnology (SIK), commissioned by FAO, proposed a clear distinction between these two expressions. More specifically, with FL are meant losses occurring upstream in the food supply chain, mainly during planting, cultivation, harvesting, processing, storage and first agricultural processing, losses usually caused by inefficiencies in the supply chain. On the other hand, FW refers to any wholesome, edible substance that is wasted, lost, degraded, or consumed by pests at any stage of the agrifood supply chain, instead of being intended for human consumption [13].

The need to analysing this issue arises if we consider the very high levels of FW in the World. In fact, in 2021, was evaluated that approximately 1.3 billion tonnes were lost or wasted: one third of food produced for human consumption was wasted and households contributed to the largest share of food waste (42%) [14]. And these data are supposed to increase considering the growth of the world population. FAO [15] estimates the world population will grow to 9.6 billion by 2050. This phenomenon will have key global impacts by causing significant rise in food demand, determining pressure on supply chains to reach higher levels of food production. Continued growth in population and consumption worldwide will increase the global demand for food for at least another 40 years, thus causing an ever more intensive use of natural resources, particularly soil, water and energy [4, 16]. Moreover, this would boost environmental concerns due to greenhouse gas emissions and probable high level of food waste generation [17–19]. Finally, FAO [20] has indicated that almost 14% of the generated food degrades before it is sold, and approximately 17% of the entire food volume is wasted at the household level.

Therefore, in this framework it is important to structure and implement adequate and responsive strategies to minimize FW production, involving not only governments and policy makers but also retailers, food producers, households at each stage of the supply chain. Going into more detail, considering that households are the most impactful players in this phenomenon, it is important to identify what are the factors influencing FW levels and consequently, to outline the most appropriate strategies for a more sustainable food system [21–24].

The objective of this study is to investigate and identify the main factors influencing FW household behaviours. The so obtained output could be useful for policy makers and stakeholders in outlining specific and sustainable strategies aiming to FW reduction. Starting from the large number of published studies on this subject [25–28], with a similar aim but focusing on specific Countries or contexts [29–32], the goal of our study is achieved through the implementation of a systematic literature review followed by a bibliometric review through the use of VOSviewer software that will allow to implement an objective and replicable bibliometric analysis. Though VOSviewer is a software for the analysis of complex phenomena in many fields [33–35], to the best of our knowledge it was never applied in the FW topic. The key feature of this software is the management of a large number of papers that are classified into clusters allowing a better analysis and summary of the results of the literature review.

The paper is structured as follow: methodology is described in Section 2, results are presented in Section 3, while the discussion is reported in Section 3. Concluding remarks about the main findings are provided in Section 4, with also some insights for future research.

## 2. Methodology

According to [36] there are 14 types of paper review methodologies. They have some steps in common, but can also differ in some features. The most common is the "narrative" literature review. It is based on less objective choices and lacks rigor and reproducibility if compared to the systematic review which, conversely, represents a more structured and reproducible methodology. The wide use of systematic reviews has been boosted by the continued growth of research. Indeed, the features of this methodology make it appropriate for evaluating and analysing a large number of documents. Mainly, its strengths are i) to be able to summarize the current state of the art ii) to highlight research gaps and lastly iii) to highlight methodological weaknesses in those studies in order to improve future research [37]. Although systematic reviews have many advantages, such reviews are susceptible to some biases (i.e. broader and less objective results). Therefore, in this study, a mixed approach was chosen to limit the effect of bias through the merging of a systematic literature review and bibliometric analysis. Indeed, bibliometric analysis uses a set of quantitative methods to measure, map and investigate the academic literature, enhancing the review with quantitative data and indicators of bibliometric activity [38].

In particular, a keyword analysis and searching was used for the literature review, while VOSviewer software was applied for following bibliometric analysis.

The above mentioned tool separates data into clusters and assigns different colours to each cluster. Other advantages for choosing VOSviewer software are the ease of use and the possibility to manage a significant number of publications.

More specifically, the first step was creating a research query with principal and ancillary keywords. Principal keywords such as "households food waste" OR “domestic food waste” were used to limit the search to papers clearly focused on this specific topic, while a set of ancillary keywords such as “attitudes”, “determinants”, “behaviour” was chosen to identify specific studies on household behaviours.

The query was developed in Scopus with the “TITLE-ABS-KEY” operator.

The reasons for choosing the Scopus database were mainly represented by the following considerations: *i*) Scopus gives relevant and reliable information on publications (also with bibliographic data), because great importance it assigned to peer review procedure [33]; *ii*) compared to Web of Science (another search engine very frequently used in bibliometric analyses), Scopus makes possible the evolution and citation analysis because it has a 20% wider coverage in time [39]; *iii*) Scopus allows direct export of data in a format supported by most of the bibliometric analysis software [33].

With the aim of evaluating the complete evolution of the research topic over time, no limitations to specific years were applied.

Considering the multidisciplinarity and cross-disciplinarity of this research issue, all subject areas were considered.

**Fig 1:**
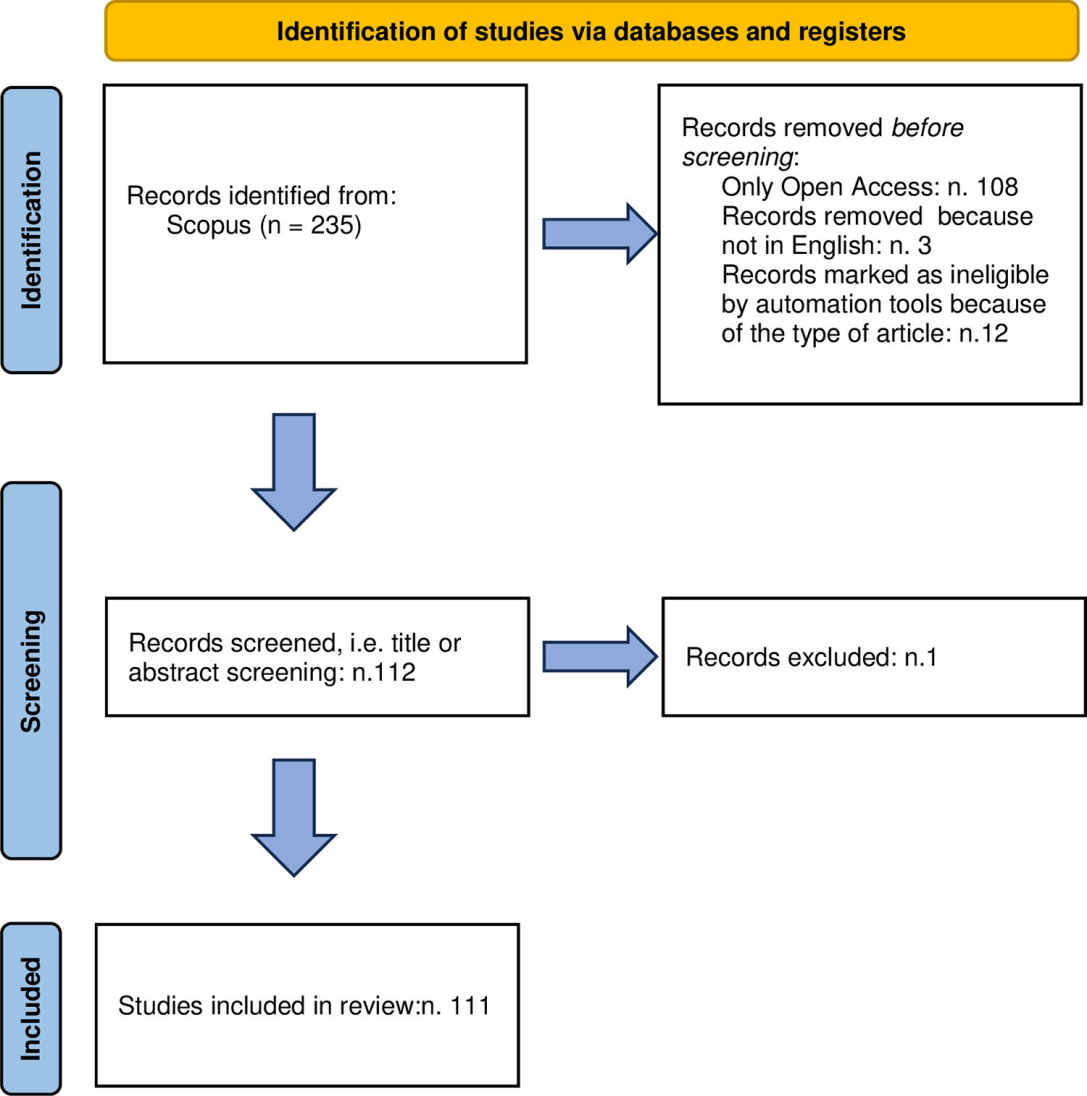
The flow chart of the articles’ selection process. Source: our elaboration base on PRISMA flow.

As shown in Fig 1, the Scopus research generated a total of 235 matching papers.

Considering the other inclusion screening criteria, such as open access sources, papers written in English language and documents published in scientific journals, at the time of the papers’ selection (22th August 2022), the useful studies were 112. Finally, after abstracts reading, as only one paper diverged from the research question, 111 papers were collected for the systematic review. Fig 1 depicts the selection route employed in this study, adhering to the PRISMA approach. This decision facilitated comprehensive monitoring of the entire paper selection process for analysis.

During the second step of the research the selected papers by means of bibliometric analysis were analysed using VOSviewer software, a powerful instrument to map and visualize network structure with bibliographic results coming from several search engines [40]. The results from bibliometric analysis and systematic review are reported in the following section.

## 3. Results and discussion

### 3.1 Results by bibliometric analysis

**Fig 2:**
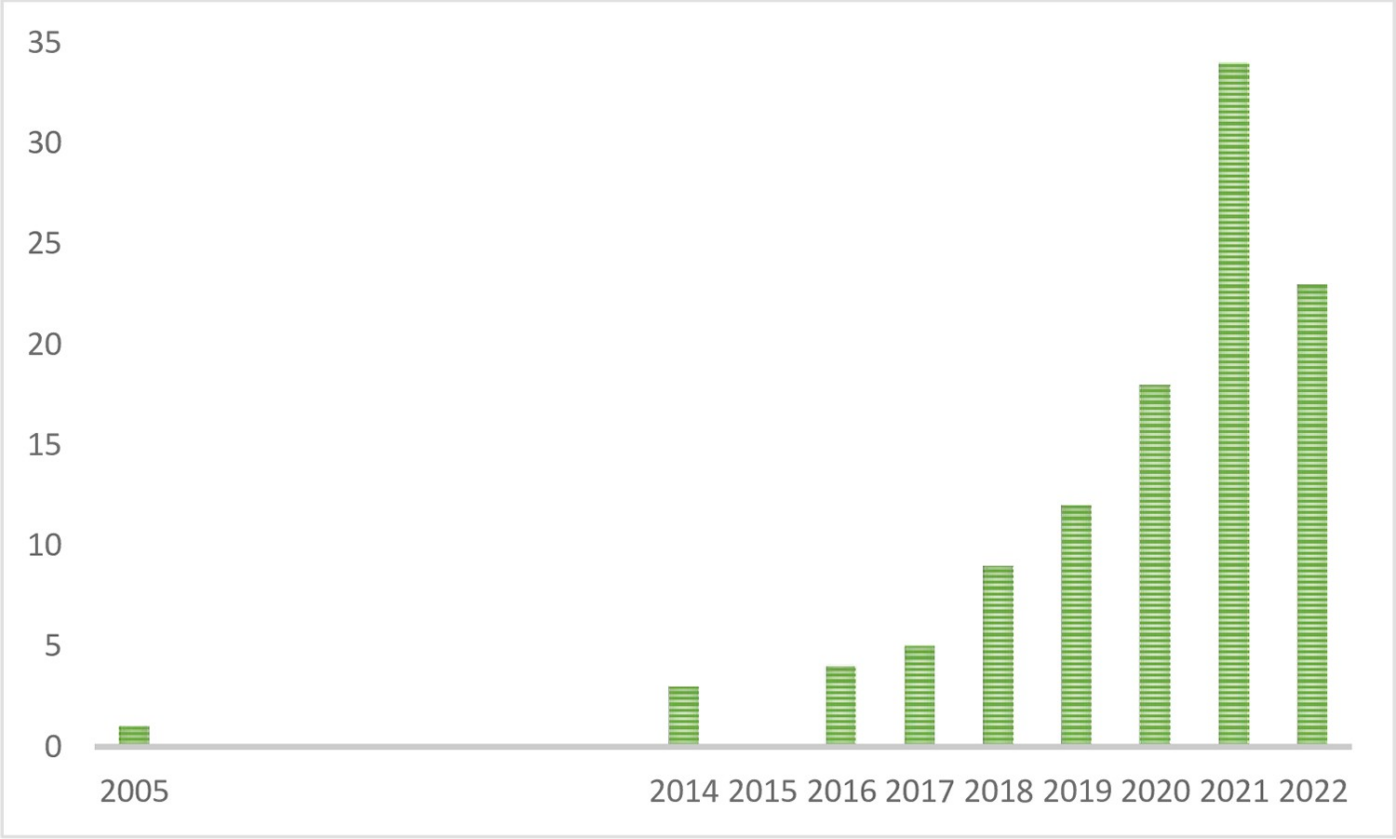
Scientific papers’ distribution over the time. Source: our elaboration.

As shown in Fig 2, reporting the distribution of 111 papers on the timeline, the selected papers were published between 2005 and 2022 (year 2022 was included, although still in progress, due to the large number of papers published).

Moreover, results show that only one paper was published in 2005 and three papers in 2014, with a publication gap of 9 years. However, this could be due to a limitation of the query or to the chosen methodology. To overcome this gap, efforts were made to include additional keywords such as ’urban food waste,’ ’city food waste,’ and ’resident food waste’ in the search criteria. However, these keywords yielded insignificant additions to the paper selection. Moreover, including these keywords could lead to papers that were not directly related to the topic, such as waste generated in restaurants, shops, or offices [41]. Therefore, these additional keywords were excluded from the search process. In this scenario, the hypothesis is that choosing only open-access papers influences the outcome. While this choice may impose certain limitations on the selection of documents, it also enhances the replicability of the study’s process and results. Given the nature of the topic and its relevance to diverse stakeholders with varying levels of interest in analysing this issue, it was deemed appropriate to focus solely on open-access papers.

Fig 2 also highlights an increase of publications starting from 2015, the year in which the 2030 Agenda for Sustainable Development was signed, emphasizing the worldwide relevance recognized to these issues.

Regarding the editorial collocation of papers dealing with FW topics, Table 1 shows the main journals in which at least two documents of the selected 111 were published.

**Table 1:** Major journals publishing studies on FW.

Journal	Number of papers
Sustainability (Switzerland)	30
Resources, Conservation and Recycling	10
Journal of Cleaner Production	8
Socio-Economic Planning Sciences	6
Appetite	5
Foods	4
Plos One	4
International Journal of Environmental Research and Public Health	3
Waste Management	3
Frontiers in Nutrition	3
Frontiers in Sustainable Food Systems	3
Sustainable Production and Consumption	2
Environmental Science and Pollution Research	2
Frontiers in Environmental Science	2

Source: our elaboration

The journal with the highest number of publications is “Sustainability” (Switzerland), probably because the research criteria included only open sources papers. It is followed by “Resources, Conservation and Recycling” having 10 papers, “Journal of Cleaner Production” with 8 studies and “Socio-Economic Planning Sciences” with 6 scientific articles.

The other journals, having 5 or less published paper on FW topic, address mainly sustainability, food and social issues, highlighting the relevance of FW at economic, social, and environmental level.

A co-occurrence analysis of keywords used by scholars was developed. This analysis focuses on the knowledge structure of a specified field exploring the links among the keywords used in the literature [42]. The minimum number of occurrences of a keyword was set to three, so considering keywords that appear at least three times together in different clusters was generated.

**Fig 3:**
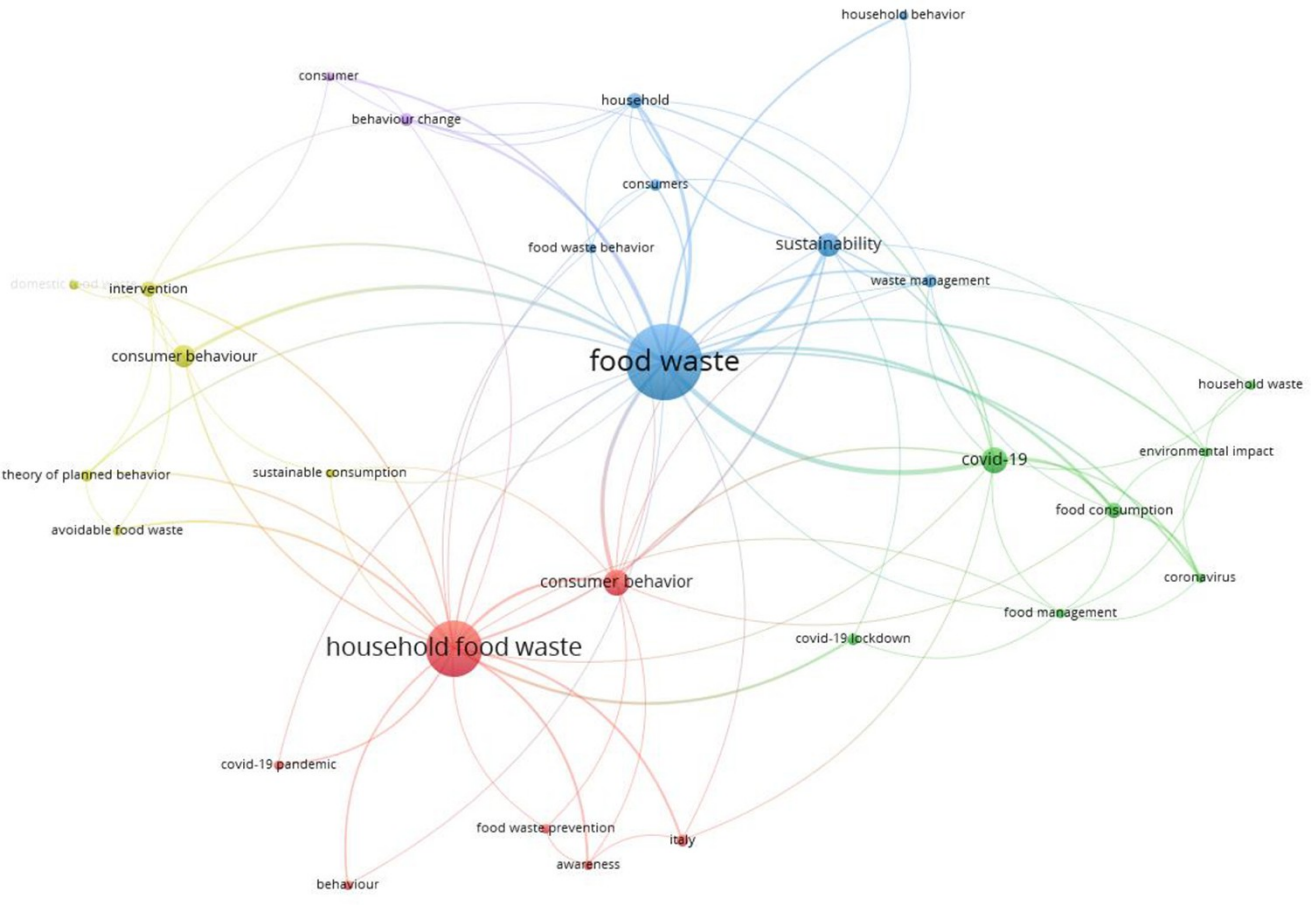
Author keyword network mapping. Source: VOSviewer elaboration.

**Fig 4:**
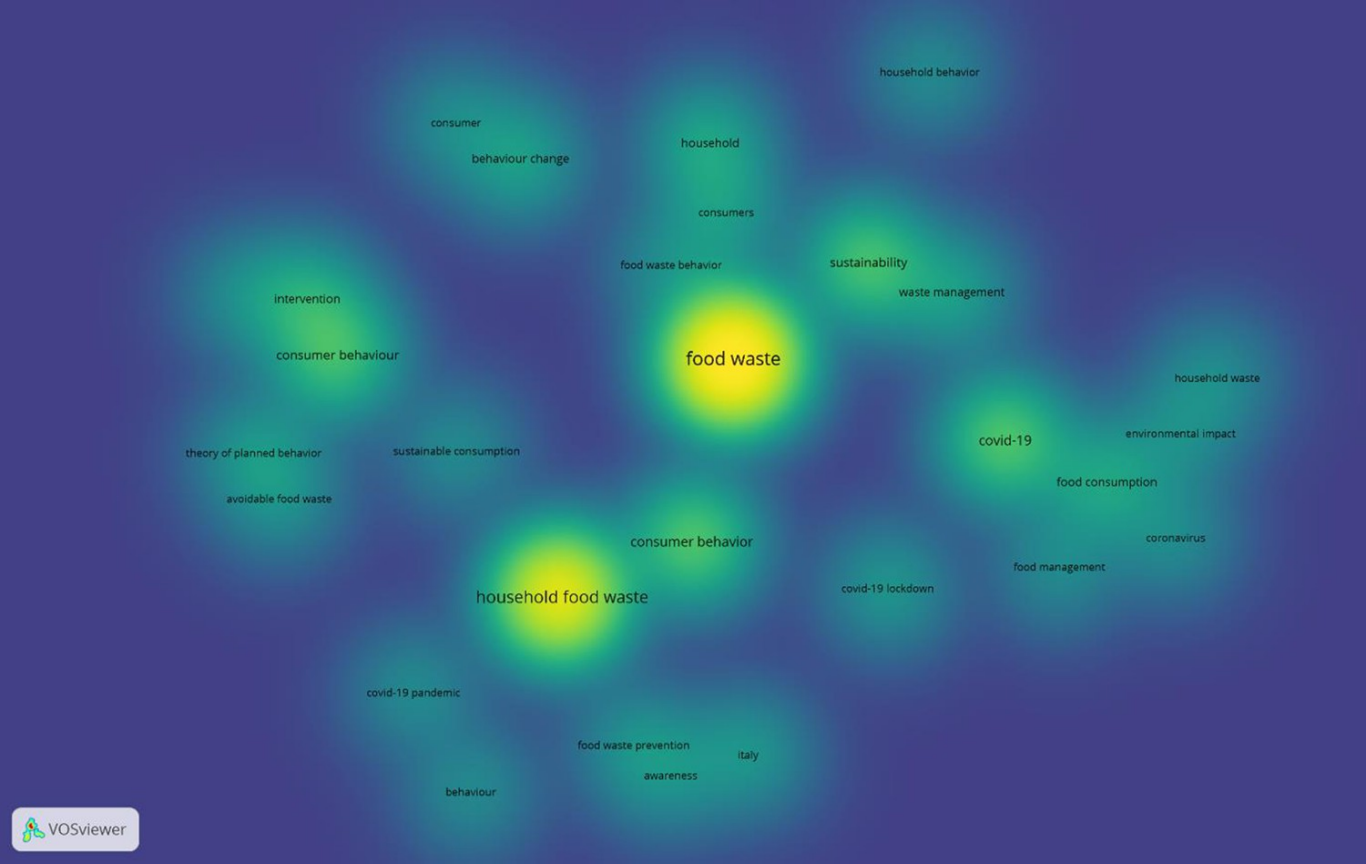
Keywords analysis- Density visualization. Source: VOSviewer elaboration.

Results show that 29 keywords out of 344 fitted the chosen criteria, so five clusters were formed. The keywords with the highest number of matches are: "food waste"; "household food waste," and "Covid-19", while words such as "intervention", "theory of planned behavior," and "environmental impact" though included, have minimal importance (Fig 3). In addition, Fig 4 shows “density visualization”, where the deep yellow indicates a higher frequency of keyword usage [43].

### 3.2 Results by VOSviewer analysis

**Fig 5:**
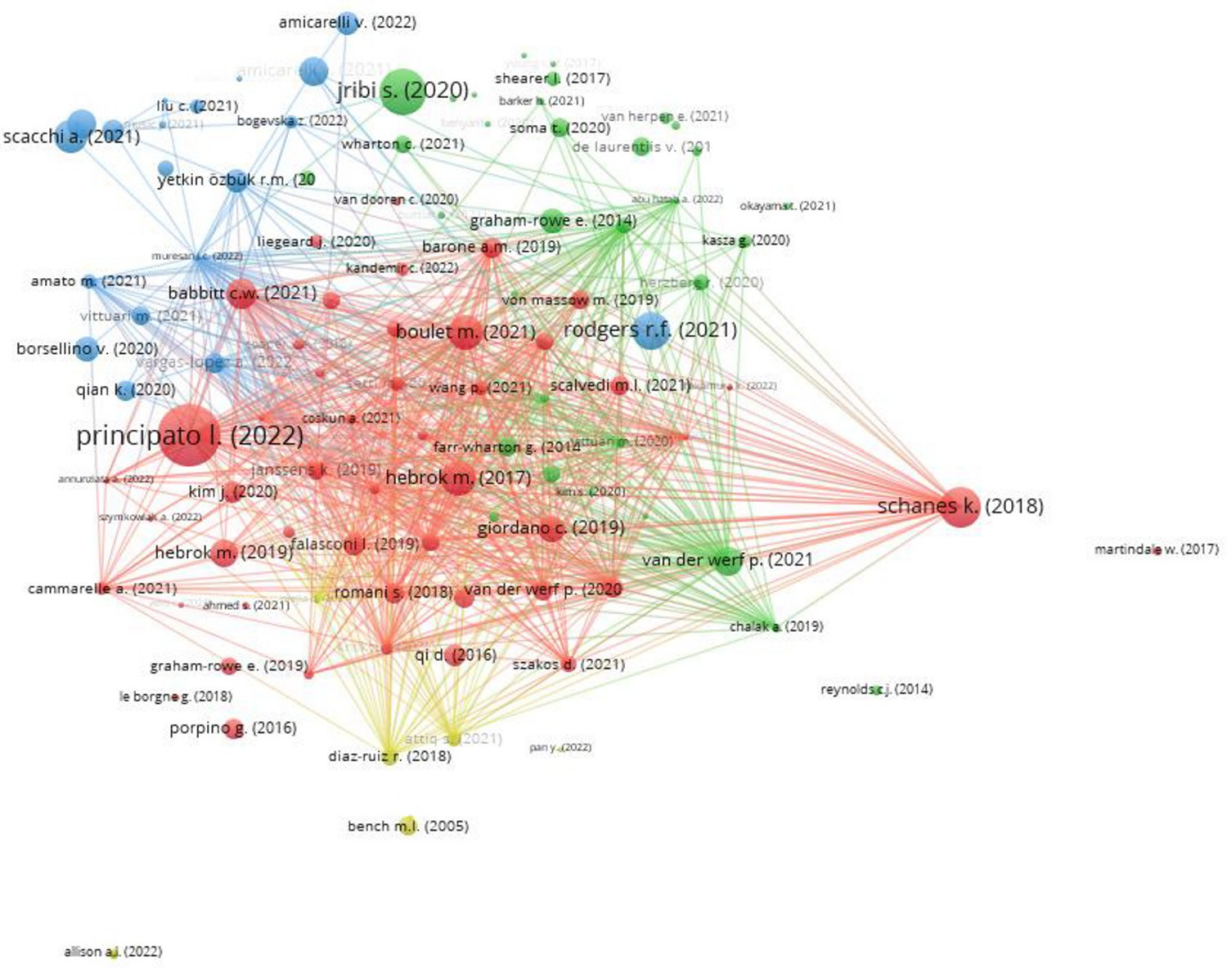
Results of the VOS analysis. Source: VOSviewer elaboration.

Fig 5 shows the graphic output of the VOS analysis. It reveals the presence of four clusters representing the streams of research within the field of FW.

From a first visual examination, it emerges that the clusters are not very distinct. Specifically, it is evident that the Red Cluster is the largest one and that it is also interconnected and overlapping with all the others, implying that some themes included in this cluster are reflected also in the other three.

The descriptive statistics related to the detected clusters are presented in Table 2, while the bibliographical data for each article are reported in Table A in S1 Appendix.

**Table 2:** Cluster descriptive statistics.

	Number of Papers	Total Citations	Total Normalised Citations	Total Citations / Number of Articles
**Red Cluster**	48	1828	396.695	38.08
**Green Cluster**	33	1090	119.570	33.03
**Blue Cluster**	20	244	178.421	12.20
**Yellow Cluster**	8	125	2,9352	15.62
**Total**	**109**			

Source: our elaboration

This analysis highlighted that some of the 111 selected documents are not connected to each other. Thus, the largest set of connected items is based on 109 documents because two documents were not linked in terms of shared references.

In general, the Red and Green clusters include the largest number of articles (respectively 48 and 33). Specifically, the Red cluster has also the largest number of total citations (No. 1828) and is the most relevant considering the Total Citations/Number of Articles ratio (48 papers received 1828 citations).

These results highlight that FW is a cited and significant field of research.

Different topics emerge from the reading of the four detected clusters, that can be summarized as follows:

- The Red cluster, titled “*Household attitudes towards FW generation”*, focuses on the analysis of items that can influence the household behaviour during food management.
- The Green cluster, titled *“Economic impacts and different types of interventions”*, focuses on the economic impact of FW.
- The Blue cluster, titled *“The impact of Covid-19”*, analyses FW during the pandemic period.
- The Yellow cluster, titled *“The environmental and social aspect”*, investigates the reduction both of food purchased and FW production.

The specific characteristics of each cluster are described below.

#### 3.2.1. Red cluster: *“Household attitudes towards FW generation”*

The red cluster contains most of the papers (n. 48) analysing behavioural factors, intentions and attitudes that may influence household FW.

Fig 5 shows that the most linked paper deals, through the use of a questionnaire, with the antecedents of FW at the household level during Covid-19 time [44–47].

This cluster analysis highlights that the most wasted products are fruits, vegetables and dairy products, because of their perishability and low perceived value [28, 48, 49].

Nevertheless the literature does not agree with the influence of variables such as gender, age, education, and income [4, 44, 50]. Probably, this discrepancy could be explained by the data collection on consumer behaviours and FW through questionnaires or interviews (23 out of 49 items) although for this topic the use of these tools is not recommended. Indeed, according to [7, 51], asking consumers to self-fill out a questionnaire could lead to significant biases, self-reported data may not agree with reality and declared attitudes may not reflect the true respondents’ behaviours.

Table 3 shows the factors that may influence the level of FW. Many of these studies highlighted behaviours that can increase or decrease FW. Meal planning and shopping lists can reduce FW [52–60], cooking or serving too much food can increase this phenomenon [26, 53, 54, 61–63] as well as frequency purchase could increase FW [22, 58, 64].

**Table 3:** The factors that may influence FW.

Factors influencing FW	Relation to FW	Authors
Cooking or serving too much food; food preferences; scarce culinary food skills; recipes for reuse	Increase	11: (Ammann et al., 2021 [61]; Boulet et al., 2021 [53]; Chakona and Shackleton, 2017 [26]; Falasconi et al., 2019 [54]; Hebrok and Heidenstrøm, 2019 [55]; Nakamura et al., 2022 [57]; Piras et al., 2021 [58]; Principato et al., 2020 [59]; Romani et al., 2018 [60]; Szakos et al., 2021 [63]; Scalvedi M.L. (2021) [65])
Planning meals and purchases with a shopping list	Increase	9: (Boulet et al., 2021 [53]; Falasconi et al., 2019 [54]; Hebrok and Heidenstrøm, 2019 [55]; Mallinson et al., 2016 [56]; Nakamura et al., 2022 [57]; Piras et al., 2021 [58]; Principato et al., 2020 [59]; Romani et al., 2018 [60]; Teng C.-C. (2021) [66])
Misreading expiration date and labels; incorrect storage of food.	Increase	9: (Babbitt et al., 2021 [67]; Falasconi et al., 2019 [54]; Hebrok and Heidenstrøm, 2019 [55]; Principato et al., 2020 [59]; Romani et al., 2018 [60]; Stancu and Lähteenmäki, 2022 [22]; Szakos et al., 2021 [63]; Liegeard J. (2020) [68]; Martindale W. (2017) [69])
In-store purchasing errors: buy too much food, offers, large packages	Increase	8: (Boulet et al., 2021 [53]; Falasconi et al., 2019 [54]; Le Borgne et al., 2018 [49]; Principato et al., 2020 [59]; Romani et al., 2018 [60]; Janssens K. (2019) [70]; Tsalis G. (2020) [71]; Kandemir C. (2022) [72])
Food safety; Healthy eating; To be a good provider;	Increase	3: (Babbitt et al., 2021 [67]; Barone et al., 2019 [73]; Boulet et al., 2021 [53])
The importance of the smell and aesthetic of food	Increase	3: (Ammann et al., 2021 [61]; Hebrok and Boks, 2017 [74]; Hebrok and Heidenstrøm, 2019 [55])
Frequency purchase	Decrease	3: (Giordano et al., 2019 [64]; Piras et al., 2021 [58]; Stancu and Lähteenmäki, 2022 [22])
Good stock organization of the kitchen or fridge	Decrease	2: (Hebrok and Boks, 2017 [74]; Hebrok and Heidenstrøm, 2019 [55])
Varieties of food available at low price	Increase	2: (Hebrok and Boks, 2017 [74]; Romani et al., 2018 [60])
To buy from large supermarket chains	Decrease	2: (Boulet et al., 2021 [53]; Principato et al., 2020 [59])
Buy in bigger cities	Decrease	2: (Nakamura et al., 2022 [57]; Principato et al., 2020 [59])
Family members eating together	Decrease	2: (Chakona and Shackleton, 2017 [26]; Hebrok and Boks, 2017 [74])
Packaging size	Increase	3: (Hebrok and Heidenstrøm, 2017 [55]; Coskun A. (2021) [75]; Oláh J. (2022) [76])
Recycling	Decrease	1: (Giordano et al., 2019 [64])
Social Capital level	Decrease	2: (Piras et al., 2021 [58]; Alattar M.A. (2021) [77])
High perceived value	Decrease	1: (Hebrok and Boks, 2017 [74])

Source: our elaboration

In addition, other motivations supporting policy makers to develop specific interventions could be detected. Among these, the most significant are: *i*) a good level of social capital of the territories allows FW to be reduced [58]; *ii*) good stock organization of the kitchen or fridge can help consumers to better manage their food stocks so avoiding wastes [74, 77]; *iii*) packaging size can influence consumer purchasing behaviour and support efficient food use [78, 79].

Regarding methodology, many papers in the red cluster used the Theory of Planned Behaviour (TPB) as a scale to assess respondents’ intentions and behaviours [80–82]. The analysis revealed that the intention to reduce FW is predicted by the individuals’ attitudes, in fact people who have a higher intention to reduce FW reports lower levels of waste [73]. These analyses show that perceived behavioural control, rather than intention, are the most important factors. In addition, some authors have tried to add other specific items, such as *i*) meal planning; *ii*) food storage behaviours; *iii*) attitude or *iv*) food management behaviours, to the TPB to better investigate the household FW phenomenon [83, 84].

#### 3.2.2. Green cluster: *“Economic impacts and different types of interventions”*

The green cluster includes 33 papers. The paper with the most normalized citations was written by [85]. Already reading this study, a different approach to FW analysis clearly appears in this cluster. In general, the green cluster papers argue that different levels of governance have to cooperate to manage the issue [86]. The “profitability”, is one of the terms introduced in this group of papers, it is expression that the economic sphere is the main as one field of investigation [87]. The economic aspects are also underlined by new suggestions for FW management, such as financial penalties or economic incentives, whereas in previous papers the focus was on behaviours to be attempted at home or in stores, without economic repercussions [88–91].

According to [92], for many consumers, financial reasons are the key motivations keys for minimizing FW, so, the focus shifts to the waste of money, unlike the red cluster, where the FW was analysed as a behavioural issue [93–96].

Another difference from red cluster is based on several suggested interventions. Some papers emphasize the need to develop not only informative activities, but linked to real actions, e.g., suggestions or demonstrations of desirable behaviours [97, 98].

Educational and awareness-raising interventions, mainly student-oriented at school, are considered successful in reducing FW, because developing awareness at young age has positive impacts on future attitudes [99, 100]. Others good practices suggested in green cluster, as alternatives to more traditional actions, are *‘nudge interventions’*. Essentially, this approach is based on the idea of adjusting the way in which options are offered to consumers. The goal then is to make the best choice more attractive. The use of this technique aims to help consumers adopt the best choice for themselves [101, 102].

From a policy perspective, the cost-effectiveness of developing these actions and the ease of use and adaptation in different contexts make the nudge a useful tool for policy makers. Indeed, this approach can also have significant results if used as a supplement to other implemented policies [103].

As seen in Fig 5 the different clusters are overlapped, hence in this group there are some factors regarding household FW. Specifically, consumer’s habits [104–106]; wrong interpretation of the expiry date increases the FW [27, 107]; low cooking skills negatively affect the reduction of FW [108, 109]; purchasing and preparing correct portions of food can decrease FW [27, 110]; the weight given to social behaviours and reputation can influence food management at the household level [86, 111–114].

#### 3.2.3. Blue cluster: *“The impact of Covid-19”*

The third cluster includes 20 items analysing the impacts of Covid-19 on FW. Many authors focused on pandemic and health restrictions effects to investigate consumers’ food management behaviour [115–121].

Results of the literature analysis are conflicting: some lifestyle attitudes (e.g., diet quality) received positive changes during the pandemic period [30, 122]; while other behaviours (e.g., impulsive shopping or panic buying) had a negative influence on FW reduction [123, 124].

To better summarize the findings, Table 4 shows the behaviours that affected the level of household FW during the pandemic.

**Table 4:** Household behaviours during Covid-19.

Factors influencing FW	FW	Authors
Increased food purchasing and consumption, improved diet quality, increased time spent cooking at home	Increase	14: (Amato et al., 2021 [25]; Amicarelli et al., 2022 [125]; Cosgrove et al., 2021 [122]; Iranmanesh et al., 2022 [126]; Laila et al., 2022 [127]; Liu et al., 2021 [42]; Music et al., 2021 [124]; Pires et al., 2020 [32]; Qian et al., 2020 [115]; Rodgers et al., 2021 [117]; Scacchi et al., 2021 [128]; Vargas-Lopez et al., 2022 [118]; Vittuari et al., 2021 [129]; Yetkin Özbük et al., 2021 [119])
Management of food stocks and leftovers	Decrease	7: (Amato et al., 2021 [25]; Amicarelli et al., 2022 [125]; Berjan et al., 2022 [123]; Cosgrove et al., 2021 [122]; Laila et al., 2022 [127]; Scacchi et al., 2021 [128]; Vittuari et al., 2021 [129])
Online food shopping	Increase	6: (Amicarelli et al., 2022 [125]; Iranmanesh et al., 2022 [126]; Laila et al., 2022 [127]; Liu et al., 2021 [42]; Pires et al., 2020 [32]; Scacchi et al., 2021 [128])
"Panic buying"	Increase	6: (Amicarelli et al., 2022 [125]; Berjan et al., 2022 [123]; Cosgrove et al., 2021 [122]; Iranmanesh et al., 2022 [126]; Music et al., 2021 [124]; Scacchi et al., 2021 [128])
Time management	Decrease	3: (Amicarelli et al., 2022 [125]; Laila et al., 2022 [127]; Vittuari et al., 2021 [129])
Smartworking; consumption of food outside the home.	Increase	2: (Amicarelli et al., 2022 [125]; Scacchi et al., 2021 [128])
Economic impact	Decrease	1: (Amicarelli et al., 2021 [125])
Use of Google searches for recipes	Decrease	1:(Scacchi et al., 2021 [128])
Emotional overeating	Increase	1:(Scacchi et al., 2021 [128])
Familiarity with domestic appliances	Decrease	1: (Amicarelli et al., 2022 [125])
Misreading expiration date and labels	Increase	1: (Berjan et al., 2022 [123])

Source: our elaboration

Results show that the increase in available time [125, 127, 129] and a more careful organization of food shopping (such as developing a shopping list) can reduce the FW [25, 32, 42, 124]. On the other hand, some behaviours can rise it as, for example, the increase in panic buying due to the difficult health situation [123, 124, 126] and emotional overeating, as food was one tool to counteract stress and anxiety management [128].

It should be noted that the different degree of virus diffusion is important in the analysis of this cluster. Many attitudes and behaviours developed differently among countries, in fact, where the pandemic was more widespread, more concern for food and food safety was developed [118, 129].

Finally, the analysis of consumer behaviour during the Covid-19 period leads to two important questions. The first one is whether the new lifestyles and related changes in food management will be permanent or not; while the second, highlighted by [125], relates the decrease in FW during the pandemic period that could be only an apparent results, but it will return as a consequence of accumulated food.

#### 3.2.4. Yellow cluster: “*The environmental and social aspect”*

Although the FW is one of the Goals within the Agenda 2030 for Sustainable Development, in this analysis issues related to social and environmental aspects emerged only in a few selected papers. Considering that the 2030 Agenda aims to increase sustainability and life quality it is surprising that only eight papers focus on environmental or social issues.

As can be seen in Fig 5 this cluster analyses some previously discussed topics, in particular the Covid-19 effects [31] and the TPB methodology to analyse consumer behaviours [130].

Focusing on the environmental side, from the reading of the yellow cluster papers, a first result rises. More precisely, it relates the huge environmental impact of FW and the consequent idea that it is more efficient to prevent FW formation rather than to develop strategies to manage FW [131]. Specifically, to this aim, European Parliament (EU, 2008/98/EC) also promotes prevention actions as the first strategy for waste management, but unfortunately, this step does not receive sufficient attention [132]. Another important output is related to the use of FW recycling devices, technologies or stations in the home gardens, because benefits of home recycling and food composting are highlighted in many papers. Strategies oriented to implement these solutions could improve not only waste management, but could promote positive economic impacts arising from the FW collection and the reduction of disposal costs by local authorities [133–135]. In this regard, according to [133] the main hindrances to household collection are: *i*) lack of awareness of the food collection program; *ii*) lack of time or space for recycling; *iii*) lack of belief in benefits for FW resulting from self-recycling; *iv*) concerns about pests, pollution, and service implementation.

The second aspect of yellow cluster papers concerns social impact: it is pointed out that FW can create social pressure lead to attitudes that reduce FW.

Specifically, according to [136], if consumers feel emotionally guilty about FW, they will adopt behaviours such as recycling or reusing. In addition, a greater sense of community leads to improved FW reduction behaviours. In contrast, if consumers do not feel community pressure to engage in the reduction of FW, they will change their behaviours. Indeed, when consumers perceive target groups that generate an amount of FW, they adapt their behaviour based on a common moral norms [130, 131].

In conclusion, the most relevant environmental and social factors in FW reduction for a true collective change as highlighted by [136, 137] are represented by the creation of social consciousness and environmental awareness. Moreover, given the importance of society and community, cooperation among stakeholders along the full path of food should be considered [138].

## 4. Concluding remarks

As widely highlighted in literature [139–141], food waste reduction, connected to other sustainability issues such as food insecurity, climate change, land degradation and economic development are identified as the most important global current challenges.

The magnitude of food waste challenge, emphasizes the importance of examining this issue, focusing, in particular, on household level [20]. Thus, the aim of this study has been to investigate and identify the main factors influencing FW household behaviours on which policy makers and stakeholders could outline specific and sustainable strategies for addressing this issue.

The bibliometric analysis allowed to look into and to cluster over 100 papers that resulted from the Scopus search to summarize the main factors and antecedents influencing household behaviours on FW generation.

The VOSviewer analysis showed the existence of four major research strands: the largest strand analyses the antecedents of behaviour during food management, including the implementation of the Theory of Planned Behaviour (TPB). Other detected topics are the economic impact of FW, the effects generated by Covid-19 pandemic in consumer behaviours, and finally, the environmental and social effects.

The review begins just from the intention to summarize the different factors that may influence FW generation at the household behaviors. Since the past Century, a wide range of factors influencing this phenomenon was identified [142, 143]. However, starting from 2015, new factors affecting household FW production and management are emerging, such as the use of QR codes and technological appliances.

Results of this study highlight that an over or inappropriate purchasing, bad storage conditions, over-preparation, portioning and cooking as well as confusion between the terms ‘‘best before” or ‘‘use by” dates are still some of the main factors affecting FW. This behaviour depends on a series of interconnected factors, mainly consumption behaviour and food patterns. Moreover, the barriers to overcome in achieving FW minimization at household level may also involve emotional or psychological aspects [144, 145]. Even the absence of economic incentives or financial sanctions may affect consumer behaviours [89, 146]. In addition, as emerged during the pandemic period, panic buying or emotional overeating can also promote FW generation [123, 128]. Moreover, the literature has shown how limited knowledge of food collection programs and limited space for adequate recycling can positively influence FW management [133].

Results of this study offer several implications and the possibility to support stakeholders and policy makers in defining more specific strategies for household FW reduction.

One possible action could be the diffusion, through the media or social networks, of motivational messages based on environmental respect to improve consumers awareness on this issue. However, to reduce the ineffectiveness of such strategy highlighted by [92], due to the "*global warming fatigue*", this communication strategy should be combined with the indication of real example behaviours that consumers could adopt or follow [146].

This overall strategy could contribute to maximize the positive impacts on FW reduction experienced during the Covid-19 pandemic best practices such as a more careful food shopping organisation, the improved cooking and food preparation skills.

In addition, as highlighted by [5, 135], FW reduction is a complex issue whose management requires the involvement of many stakeholders capable to support policymakers in defining an effective and long-running strategy at the local level. In this regard [67] argue that a systemic view is needed for the issue management so that consumers can be involved and develop long-lasting behavioural changes.

From the environmental side, a successful vision for this challenge could be represented by the systemic approach of circular economy [147] based on the idea of regenerating and producing value even by the reuse and readmission of biological nutrients into the supply chain, just as indicated by the *butterfly graph* proposed by Boulding (1966).

Finally, to guarantee a real effectiveness of the different actions and strategies, the harmonization of the multifaceted and fragmented policy framework developed by several global organizations (such as the Food and Agriculture Organization and SDGs of the United Nations, European Commission, and the World Health Organization) would be desirable.

However, some potential limitations of the study depend on the query keywords or the choice to analyse only Scopus results that may have created biases.

Future research could expand and improve the search indicators and the use of multiple search engines and, given the complex nature of the issue, a multidisciplinary approach would be recommended.

## Supporting information

S1 File The file provides the whole dataset as an excel file derived from VOSviewer elaboration. (XLSX)

S1 Appendix (DOCX)
