# Supplementary material for: Factors affecting food waste: A bibliometric review on the household behaviors
Source: PLoS One. 2023 Jul 28;18(7):e0289323. doi: 10.1371/journal.pone.0289323 (PMC10381066; doi:10.1371/journal.pone.0289323)
Supplement: S1 Appendix — (DOCX) [file pone.0289323.s002.docx]

**Appendix**

**Table A-all the papers considered in the study**

| **VOS Label** | **Authors** | **Title** | **Journal** | **Year** | **Cluster** | **Citations** | **Norm.Citations** |
| --- | --- | --- | --- | --- | --- | --- | --- |
| Principato L. (2022) | Principato L.; Secondi L.; Cicatiello C.; Mattia G. | Caring more about food: the unexpected positive effect of the Covid-19 lockdown on household food management and waste | Socio-Economic Planning Sciences, 82 | 2022 | Red | 62 | 141.188 |
| Boulet M. (2021) | Boulet M.; Hoek A.C.; Raven R. | Towards a multi-level framework of household food waste and consumer behaviour: untangling spaghetti soup | Appetite, 156 | 2021 | Red | 34 | 40.203 |
| Hebrok M. (2017) | Hebrok M.; Boks C. | Household food waste: drivers and potential intervention points for design – an extensive review | Journal of Cleaner Production, 151, 380-392 | 2017 | Red | 204 | 39.231 |
| Babbitt C.W. (2021) | Babbitt C.W.; Babbitt G.A.; Oehman J.M. | Behavioral impacts on residential food provisioning, use, and waste during the Covid-19 pandemic | Sustainable Production and Consumption, 28, 315-325 | 2021 | Red | 24 | 28.378 |
| Hebrok M. (2019) | Hebrok M.; Heidenstrøm N. | Contextualising food waste prevention - decisive moments within everyday practices | Journal of Cleaner Production, 210, 1435-1448 | 2019 | Red | 63 | 23.774 |
| Qi D. (2016) | Qi D.; Roe B.E. | Household food waste: multivariate regression and principal components analyses of awareness and attitudes among U.S. consumers | Plos One, 11(7) | 2016 | Red | 136 | 14.624 |
| Van Der Werf P. (2020) | Van Der Werf P.; Seabrook J.A.; Gilliland J.A. | Food for thought: comparing self-reported versus curbside measurements of household food wasting behavior and the predictive capacity of behavioral determinants | Waste Management, 101, 18-27 | 2020 | Red | 33 | 12.665 |
| Barone A.M. (2019) | Barone A.M.; Grappi S.; Romani S. | The road to food waste is paved with good intentions: when consumers' goals inhibit the minimization of household food waste | Resources, Conservation and Recycling, 149, 97-105 | 2019 | Red | 32 | 12.075 |
| Porpino G. (2016) | Porpino G. | Household food waste behavior: avenues for future research | Journal of the Association for Consumer Research, 1(1), 41-51 | 2016 | Red | 110 | 11.828 |
| Ammann J. (2021) | Ammann J.; Osterwalder O.; Siegrist M.; Hartmann C.; Egolf A. | Comparison of two measures for assessing the volume of food waste in Swiss households | Resources, Conservation and Recycling, 166 | 2021 | Red | 10 | 11.824 |
| Mallinson L.J. (2016) | Mallinson L.J.; Russell J.M.; Barker M.E. | Attitudes and behaviour towards convenience food and food waste in the United Kingdom | Appetite, 103, 17-28 | 2016 | Red | 106 | 11.398 |
| Romani S. (2018) | Romani S.; Grappi S.; Bagozzi R.P.; Barone A.M. | Domestic food practices: a study of food management behaviors and the role of food preparation planning in reducing waste | Appetite, 121, 215-227 | 2018 | Red | 97 | 11.124 |
| Scalvedi M.L. (2021) | Scalvedi M.L.; Rossi L. | Comprehensive measurement of Italian domestic food waste in a European framework | Sustainability (Switzerland), 13(3), 1-17 | 2021 | Red | 9 | 10.642 |
| Janssens K. (2019) | Janssens K.; Lambrechts W.; Van Osch A.; Semeijn J. | How consumer behavior in daily food provisioning affects food waste at household level in The Netherlands | Foods, 8(10) | 2019 | Red | 28 | 10.566 |
| Von Massow M. (2019) | Von Massow M.; Parizeau K.; Gallant M.; Wickson M.; Haines J.; Ma D.W.L.; Wallace A.; Carroll N.; Duncan A.M. | Valuing the multiple impacts of household food waste | Frontiers in Nutrition, 6 | 2019 | Red | 27 | 10.189 |
| Schanes K. (2018) | Schanes K.; Dobernig K.; Gözet B. | Food waste matters - a systematic review of household food waste practices and their policy implications | Journal of Cleaner Production, 182, 978-991 | 2018 | Red | 483 | 5.539 |
| Falasconi L. (2019) | Falasconi L.; Cicatiello C.; Franco S.; Segrè A.; Setti M.; Vittuari M. | Such a shame! A study on self-perception of household food waste | Sustainability (Switzerland), 11(1) | 2019 | Red | 38 | 1.434 |
| Giordano C. (2019) | Giordano C.; Alboni F.; Falasconi L. | Quantities, determinants, and awareness of households' food waste in Italy: a comparison between diary and questionnaires quantities | Sustainability (Switzerland), 11(12) | 2019 | Red | 53 | 2 |
| Kim J. (2020) | Kim J.; Rundle-Thiele S.; Knox K.; Burke K.; Bogomolova S. | Consumer perspectives on household food waste reduction campaigns | Journal of Cleaner Production, 243 | 2020 | Red | 37 | 1,42 |
| Stancu V. (2022) | Stancu V.; Lähteenmäki L. | Consumer-related antecedents of food provisioning behaviors that promote food waste | Food Policy, 108 | 2022 | Red | 4 | 0,9109 |
| Graham-Rowe E. (2019) | Graham-Rowe E.; Jessop D.C.; Sparks P. | Self-affirmation theory and pro-environmental behaviour: promoting a reduction in household food waste | Journal of Environmental Psychology, 62, 124-132 | 2019 | Red | 24 | 0,9057 |
| Piras S. (2021) | Piras S.; Pancotto F.; Righi S.; Vittuari M.; Setti M. | Community social capital and status: the social dilemma of food waste | Ecological Economics, 183 | 2021 | Red | 7 | 0,8277 |
| Tsalis G. (2021) | Tsalis G.; Jensen B.B.; Wakeman S.W.; Aschemann-Witzel J. | Promoting food for the trash bin? A review of the literature on retail price promotions and household-level food waste | Sustainability (Switzerland), 13(7) | 2021 | Red | 7 | 0,8277 |
| Szakos D. (2021) | Szakos D.; Szabó-Bódi B.; Kasza G. | Consumer awareness campaign to reduce household food waste based on structural equation behavior modeling in Hungary | Environmental Science and Pollution Research, 28(19), 24580-24589 | 2021 | Red | 6 | 0,7095 |
| Annunziata A. (2020) | Annunziata A.; Agovino M.; Ferraro A.; Mariani A. | Household food waste: a case study in Southern Italy | Sustainability (Switzerland), 12(4) | 2020 | Red | 18 | 0,6908 |
| Wang P. (2021) | Wang P.; Mccarthy B.; Kapetanaki A.B. | To be ethical or to be good? The impact of ‘good provider’ and moral norms on food waste decisions in two countries | Global Environmental Change, 69 | 2021 | Red | 5 | 0,5912 |
| Setti M. (2018) | Setti M.; Banchelli F.; Falasconi L.; Segrè A.; Vittuari M. | Consumers’ food cycle and household waste. When behaviors matter | Journal of Cleaner Production, 185, 694-706 | 2018 | Red | 49 | 0,5619 |
| Liegeard J. (2020) | Liegeard J.; Manning L. | Use of intelligent applications to reduce household food waste | Critical Reviews in Food Science and Nutrition, 60(6), 1048-1061 | 2020 | Red | 13 | 0,4989 |
| Cammarelle A. (2021) | Cammarelle A.; Viscecchia R.; Bimbo F. | Intention to purchase active and intelligent packaging to reduce household food waste: evidence from Italian consumers | Sustainability (Switzerland), 13(8) | 2021 | Red | 4 | 0,473 |
| Kandemir C. (2022) | Kandemir C.; Reynolds C.; Tom Q.; Fisher K.; Devine R.; Herszenhorn E.; Koh S.C.L.; Evans D. | Using discrete event simulation to explore food wasted in the home | Journal of Simulation, 16(4), 415-435 | 2022 | Red | 2 | 0,4554 |
| Kritikou T. (2021) | Kritikou T.; Panagiotakos D.; Abeliotis K.; Lasaridi K. | Investigating the determinants of Greek households food waste prevention behaviour | Sustainability (Switzerland), 13(20) | 2021 | Red | 3 | 0,3547 |
| Alattar M.A. (2021) | Alattar M.A.; Morse J.L. | Poised for change: university students are positively disposed toward food waste diversion and decrease individual food waste after programming | Foods, 10(3) | 2021 | Red | 3 | 0,3547 |
| Ellison B. (2018) | Ellison B.; Lusk J.L. | Examining household food waste decisions: a vignette approach | Applied Economic Perspectives and Policy, 40(4), 613-631 | 2018 | Red | 26 | 0,2982 |
| Portugal T. (2020) | Portugal T.; Freitas S.; Cunha L.M.; Rocha A.M.C.N. | Evaluation of determinants of food waste in family households in the greater Porto area based on self- reported consumption practices | Sustainability (Switzerland), 12(21), 1-12 | 2020 | Red | 7 | 0,2687 |
| Cooper K.A. (2018) | Cooper K.A.; Quested T.E.; Lanctuit H.; Zimmermann D.; Espinoza-Orias N.; Roulin A. | Nutrition in the bin: a nutritional and environmental assessment of food wasted in the UK | Frontiers in Nutrition, 5 | 2018 | Red | 21 | 0,2408 |
| Teng C.-C. (2021) | Teng C.-C.; Chih C.; Yang W.-J.; Chien C.-H. | Determinants and prevention strategies for household food waste: an exploratory study in Taiwan | Foods, 10(10) | 2021 | Red | 2 | 0,2365 |
| Coskun A. (2021) | Coskun A. | Identification of different user types for designing household food waste interventions | International Journal of Sustainable Engineering, 14(4), 609-617 | 2021 | Red | 2 | 0,2365 |
| Chakona G. (2017) | Chakona G.; Shackleton C.M. | Local setting influences the quantity of household food waste in mid-sized south African towns | Plos One, 12(12) | 2017 | Red | 11 | 0,2115 |
| Van Dooren C. (2020) | Van Dooren C.; Mensink F.; Eversteijn K.; Schrijnen M. | Development and evaluation of the eetmaatje measuring cup for rice and pasta as an intervention to reduce food waste | Frontiers in Nutrition, 6 | 2020 | Red | 5 | 0,1919 |
| Martindale W. (2017) | Martindale W. | Conference on 'sustainable food consumption' the potential of food preservation to reduce food waste | Proceedings of the Nutrition Society, 76(1), 28-33 | 2017 | Red | 9 | 0,1731 |
| Le Borgne G. (2018) | Le Borgne G.; Sirieix L.; Costa S. | Perceived probability of food waste: influence on consumer attitudes towards and choice of sales promotions | Journal of Retailing and Consumer Services, 42, 11-21 | 2018 | Red | 13 | 0,1491 |
| Ahmed S. (2021) | Ahmed S.; Stewart A.; Smith E.; Warne T.; Byker Shanks C. | Consumer perceptions, behaviors, and knowledge of food waste in a rural American state | Frontiers in Sustainable Food Systems, 5 | 2021 | Red | 1 | 0,1182 |
| Bretter C. (2022) | Bretter C.; Unsworth K.L.; Russell S.V.; Quested T.E.; Doriza A.; Kaptan G. | Don't put all your eggs in one basket: testing an integrative model of household food waste | Resources, Conservation and Recycling, 185 | 2022 | Red | 0 | 0 |
| Annunziata A. (2022) | Annunziata A.; Muca F.L.; Mariani A. | Preventing household food waste in Italy: a segmentation of the population and suggestions for action | Sustainability (Switzerland), 14(12) | 2022 | Red | 0 | 0 |
| Szymkowiak A. (2022) | Szymkowiak A.; Borusiak B.; Pierański B.; Kotyza P.; Smutka L. | Household food waste: the meaning of product’s attributes and food-related lifestyle | Frontiers in Environmental Science, 10 | 2022 | Red | 0 | 0 |
| Oláh J. (2022) | Oláh J.; Kasza G.; Szabó-Bódi B.; Szakos D.; Popp J.; Lakner Z. | Household food waste research: the current state of the art and a guided tour for further development | Frontiers in Environmental Science, 10 | 2022 | Red | 0 | 0 |
| Nakamura K. (2022) | Nakamura K.; Kojima D.; Ando M. | What reduces household food waste in Japan? Nation-wide and region-specific contributing factors in urban and rural areas | Sustainability (Switzerland), 14(6) | 2022 | Red | 0 | 0 |
| Weis C. (2021) | Weis C.; Narang A.; Rickard B.; Souza-Monteiro D.M. | Effects of date labels and freshness indicators on food waste patterns in the united states and the united kingdom | Sustainability (Switzerland), 13(14) | 2021 | Red | 0 | 0 |
| Jribi S. (2020) | Ccci S.; Ben Ismail H.; Doggui D.; Debbabi H. | Covid-19 virus outbreak lockdown: what impacts on household food wastage? | Environment, Development and Sustainability, 22(5), 3939-3955 | 2020 | Green | 196 | 75.224 |
| Van Der Werf P. (2021) | Van Der Werf P.; Seabrook J.A.; Gilliland J.A. | “Reduce food waste, save money”: testing a novel intervention to reduce household food waste | Environment and Behavior, 53(2), 151-183 | 2021 | Green | 22 | 26.014 |
| Graham-Rowe E. (2014) | Graham-Rowe E.; Jessop D.C.; Sparks P. | Identifying motivations and barriers to minimising household food waste | Resources, Conservation and Recycling, 84, 15-23 | 2014 | Green | 353 | 18.322 |
| Soma T. (2020) | Soma T.; Li B.; Maclaren V. | Food waste reduction: a test of three consumer awareness interventions | Sustainability (Switzerland), 12(3), 1-19 | 2020 | Green | 26 | 0,9979 |
| De Laurentiis V. (2018) | De Laurentiis V.; Corrado S.; Sala S. | Quantifying household waste of fresh fruit and vegetables in the EU | Waste Management, 77, 238-251 | 2018 | Green | 85 | 0,9748 |
| Farr-Wharton G. (2014) | Farr-Wharton G.; Foth M.; Choi J.H..-J. | Identifying factors that promote consumer behaviours causing expired domestic food waste | Journal of Consumer Behaviour, 13(6), 393-402 | 2014 | Green | 181 | 0,9394 |
| Fanelli R.M. (2019) | Fanelli R.M. | Using causal maps to analyse the major root causes of household food waste: results of a survey among people from central and southern Italy | Sustainability (Switzerland), 11(4) | 2019 | Green | 24 | 0,9057 |
| Wharton C. (2021) | Wharton C.; Vizcaino M.; Berardy A.; Opejin A. | Waste watchers: a food waste reduction intervention among households in Arizona | Resources, Conservation and Recycling, 164 | 2021 | Green | 7 | 0,8277 |
| Herzberg R. (2020) | Herzberg R.; Schmidt T.G.; Schneider F. | Characteristics and determinants of domestic food waste: a representative diary study across Germany | Sustainability (Switzerland), 12(11) | 2020 | Green | 20 | 0,7676 |
| Elimelech E. (2019) | Elimelech E.; Ert E.; Ayalon O. | Exploring the drivers behind self-reported and measured food wastage | Sustainability (Switzerland), 11(20) | 2019 | Green | 20 | 0,7547 |
| Shearer L. (2017) | Shearer L.; Gatersleben B.; Morse S.; Smyth M.; Hunt S. | A problem unstuck? Evaluating the effectiveness of sticker prompts for encouraging household food waste recycling behaviour | Waste Management, 60, 164-172 | 2017 | Green | 33 | 0,6346 |
| Sharp E.L. (2021) | Sharp E.L.; Haszard J.; Egli V.; Roy R.; Morenga L.T.; Teunissen L.; Decorte P.; Cuykx I.; De Backer C.; Gerritsen S. | Less food wasted? Changes to New Zealanders’ household food waste and related behaviours due to the 2020 Covid-19 lockdown | Sustainability (Switzerland), 13(18) | 2021 | Green | 5 | 0,5912 |
| Kasza G. (2020) | Kasza G.; Dorkó A.; Kunszabó A.; Szakos D. | Quantification of household food waste in Hungary: a replication study using the fusions methodology | Sustainability (Switzerland), 12(8), 1-14 | 2020 | Green | 12 | 0,4606 |
| Van Herpen E. (2021) | Van Herpen E.; De Hooge I.E.; De Visser-Amundson A.; Kleijnen M.P. | Take it or leave it: how an opt-out strategy for doggy bags affects consumer food waste behavior and restaurant evaluations | Journal of Cleaner Production, 325 | 2021 | Green | 3 | 0,3547 |
| Grainger M.J. (2018) | Grainger M.J.; Aramyan L.; Logatcheva K.; Piras S.; Righi S.; Setti M.; Vittuari M.; Stewart G.B. | The use of systems models to identify food waste drivers | Global Food Security, 16, 1-8 | 2018 | Green | 22 | 0,2523 |
| Vittuari M. (2020) | Vittuari M.; Falasconi L.; Masotti M.; Piras S.; Segrè A.; Setti M. | Not in my bin': consumer's understanding and concern of food waste effects and mitigating factors | Sustainability (Switzerland), 12(14) | 2020 | Green | 6 | 0,2303 |
| Zhang P. (2020) | Zhang P.; Zhang D.; Cheng S. | The effect of consumer perception on food waste behavior of urban households in China | Sustainability (Switzerland), 12(14) | 2020 | Green | 6 | 0,2303 |
| Reynolds C.J. (2014) | Reynolds C.J.; Mavrakis V.; Davison S.; Høj S.B.; Vlaholias E.; Sharp A.; Thompson K.; Ward P.; Coveney J.; Piantadosi J.; Boland J.; Dawson D. | Estimating informal household food waste in developed countries: the case of Australia | Waste Management and Research, 32(12), 1254-1258 | 2014 | Green | 44 | 0,2284 |
| Woolley E. (2022) | Woolley E.; Luo Z.; Jellil A.; Simeone A. | A data driven approach to reducing household food waste | Sustainable Production and Consumption, 29, 600-613 | 2022 | Green | 1 | 0,2277 |
| Piras S. (2022) | Piras S.; Righi S.; Setti M.; Koseoglu N.; Grainger M.J.; Stewart G.B.; Vittuari M. | From social interactions to private environmental behaviours: the case of consumer food waste | Resources, Conservation and Recycling, 176 | 2022 | Green | 1 | 0,2277 |
| Chalak A. (2019) | Chalak A.; Abiad M.G.; Diab M.; Nasreddine L. | The determinants of household food waste generation and its associated caloric and nutrient losses: the case of Lebanon | Plos One, 14(12) | 2019 | Green | 5 | 0,1887 |
| Niles M.T. (2020) | Niles M.T. | Majority of rural residents compost food waste: policy and waste management implications for rural regions | Frontiers in Sustainable Food Systems, 3 | 2020 | Green | 4 | 0,1535 |
| Barker H. (2021) | Barker H.; Shaw P.J.; Richards B.; Clegg Z.; Smith D. | What nudge techniques work for food waste behaviour change at the consumer level? A systematic review | Sustainability (Switzerland), 13(19) | 2021 | Green | 1 | 0,1182 |
| Okayama T. (2021) | Okayama T.; Watanabe K.; Yamakawa H. | Sorting analysis of household food waste—development of a methodology compatible with the aims of SDG12.3 | Sustainability (Switzerland), 13(15) | 2021 | Green | 1 | 0,1182 |
| Zeineddine M. (2021) | Zeineddine M.; Kharroubi S.; Chalak A.; Hassan H.; Abiad M.G. | Post-consumer food waste generation while dining out: a close-up view | Plos One, 16(6 June) | 2021 | Green | 1 | 0,1182 |
| Buttlar B. (2021) | Buttlar B.; Löwenstein L.; Geske M.-S.; Ahlmer H.; Walther E. | Love food, hate waste? Ambivalence towards food fosters people’s willingness to waste food | Sustainability (Switzerland), 13(7) | 2021 | Green | 1 | 0,1182 |
| Kim S. (2020) | Kim S.; Lee S.H. | Examining household food waste behaviors and the determinants in Korea using new questions in a national household survey | Sustainability (Switzerland), 12(20), 1-24 | 2020 | Green | 3 | 0,1151 |
| Benyam A. (2020) | Benyam A.; Rolfe J.; Kinnear S. | Willingness to pay for a domestic food waste diversion policy option in regional Queensland, Australia | Journal of Cleaner Production, 270 | 2020 | Green | 2 | 0,0768 |
| Hazuchová N. (2019) | Hazuchová N.; Tuzová M.; Macková M.; Stávková J. | Household food waste behaviour: subjective and objective evidence | Potravinarstvo Slovak Journal of Food Sciences, 13(1), 784792 | 2019 | Green | 2 | 0,0755 |
| Young C.W. (2017) | Young C.W.; Russell S.V.; Barkemeyer R. | Social media is not the ‘silver bullet’ to reducing household food waste, a response to Grainger and Stewart (2017) | Resources, Conservation and Recycling, 122, 405-406 | 2017 | Green | 3 | 0,0577 |
| Eaton E. (2022) | Eaton E.; Hunt A.; Di Leo A.; Black D.; Frost G.; Hargreaves S. | What are the environmental benefits and costs of reducing food waste? Bristol as a case study in the waste few urban living lab project | Sustainability (Switzerland), 14(9) | 2022 | Green | 0 | 0 |
| Abu Hatab A. (2022) | Abu Hatab A.; Tirkaso W.T.; Tadesse E.; Lagerkvist C.-J. | An extended integrative model of behavioural prediction for examining households’ food waste behaviour in Addis Ababa, Ethiopia | Resources, Conservation and Recycling, 179 | 2022 | Green | 0 | 0 |
| Lim V. (2021) | Lim V.; Bartram L.; Funk M.; Rauterberg M. | Eco-feedback for food waste reduction in a student residence | Frontiers in Sustainable Food Systems, 5 | 2021 | Green | 0 | 0 |
| Scacchi A. (2021) | Scacchi A.; Catozzi D.; Boietti E.; Bert F.; Siliquini R. | Covid-19 lockdown and self-perceived changes of food choice, waste, impulse buying and their determinants in Italy: quarantena, a cross-sectional study | Foods, 10(2) | 2021 | Blue | 29 | 34.291 |
| Amicarelli V. (2021) | Amicarelli V.; Tricase C.; Spada A.; Bux C. | Households’ food waste behavior at local scale: a cluster analysis after the Covid‐19 lockdown | Sustainability (Switzerland), 13(6) | 2021 | Blue | 22 | 26.014 |
| Cosgrove K. (2021) | Cosgrove K.; Vizcaino M.; Wharton C. | Covid-19-related changes in perceived household food waste in the united states: a cross-sectional descriptive study | International Journal of Environmental Research and Public Health, 18(3), 1-11 | 2021 | Blue | 22 | 26.014 |
| Borsellino V. (2020) | Borsellino V.; Kaliji S.A.; Schimmenti E. | Covid-19 drives consumer behaviour and agro-food markets towards healthier and more sustainable patterns | Sustainability (Switzerland), 12(20), 1-26 | 2020 | Blue | 48 | 18.422 |
| Amicarelli V. (2022) | Amicarelli V.; Lagioia G.; Sampietro S.; Bux C. | Has the Covid-19 pandemic changed food waste perception and behavior? Evidence from Italian consumers | Socio-Economic Planning Sciences, 82 | 2022 | Blue | 7 | 15.941 |
| Yetkin Özbük R.M. (2022) | Yetkin Özbük R.M.; Coşkun A.; Filimonau V. | The impact of Covid-19 on food management in households of an emerging economy | Socio-Economic Planning Sciences, 82 | 2022 | Blue | 7 | 15.941 |
| Berjan S. (2022) | Berjan S.; Vaško Ž.; Ben Hassen T.; El Bilali H.; Allahyari M.S.; Tomić V.; Radosavac A. | Assessment of household food waste management during the Covid-19 pandemic in Serbia: a cross-sectional online survey | Environmental Science and Pollution Research, 29(8), 11130-11141 | 2022 | Blue | 6 | 13.663 |
| Qian K. (2020) | Qian K.; Javadi F.; Hiramatsu M. | Influence of the Covid-19 pandemic on household food waste behavior in Japan | Sustainability (Switzerland), 12(23), 1-14 | 2020 | Blue | 31 | 11.898 |
| Vargas-Lopez A. (2022) | Vargas-Lopez A.; Cicatiello C.; Principato L.; Secondi L. | Consumer expenditure, elasticity and value of food waste: a quadratic almost ideal demand system for evaluating changes in Mexico during Covid-19 | Socio-Economic Planning Sciences, 82 | 2022 | Blue | 5 | 11.386 |
| Rodgers R.F. (2021) | Rodgers R.F.; Lombardo C.; Cerolini S.; Franko D.L.; Omori M.; Linardon J.; Guillaume S.; Fischer L.; Tyszkiewicz M.F. | “Waste not and stay at home” evidence of decreased food waste during the Covid-19 pandemic from the U.S. and Italy | Appetite, 160 | 2021 | Blue | 41 | 4.848 |
| Vittuari M. (2021) | Vittuari M.; Masotti M.; Iori E.; Falasconi L.; Gallina Toschi T.; Segrè A. | Does the Covid-19 external shock matter on household food waste? The impact of social distancing measures during the lockdown | Resources, Conservation and Recycling, 174 | 2021 | Blue | 8 | 0,9459 |
| Laila A. (2022) | Laila A.; Von Massow M.; Bain M.; Parizeau K.; Haines J. | Impact of Covid-19 on food waste behaviour of families: results from household waste composition audits | Socio-Economic Planning Sciences, 82 | 2022 | Blue | 3 | 0,6832 |
| Liu C. (2021) | Liu C.; Bunditsakulchai P.; Zhuo Q. | Impact of Covid-19 on food and plastic waste generated by consumers in Bangkok | Sustainability (Switzerland), 13(16) | 2021 | Blue | 5 | 0,5912 |
| Amato M. (2021) | Amato M.; Verneau F.; Coppola A.; La Barbera F. | Domestic food waste and Covid-19 concern: an application of the Theory of Planned Behaviour | Sustainability (Switzerland), 13(15) | 2021 | Blue | 5 | 0,5912 |
| Bogevska Z. (2022) | Bogevska Z.; Berjan S.; El Bilali H.; Sadegh Allahyari M.; Radosavac A.; Davitkovska M. | Exploring food shopping, consumption and waste habits in north Macedonia during the Covid-19 pandemic | Socio-Economic Planning Sciences, 82 | 2022 | Blue | 2 | 0,4554 |
| Music J. (2021) | Music J.; Charlebois S.; Spiteri L.; Farrell S.; Griffin A. | Increases in household food waste in Canada as a result of Covid-19: an exploratory study | Sustainability (Switzerland), 13(23) | 2021 | Blue | 1 | 0,1182 |
| Pires I.M. (2020) | Pires I.M.; Fernández-Zamudio M.Á.; Vidal-Mones B.; Martins R.B. | The impact of Covid-19 lockdown on Portuguese households’ food waste behaviors | Human Ecology Review, 26(1), 59-69 | 2020 | Blue | 2 | 0,0768 |
| Iranmanesh M. (2022) | Iranmanesh M.; Ghobakhloo M.; Nilashi M.; Tseng M.-L.; Senali M.G.; Abbasi G.A. | Impacts of the Covid-19 pandemic on household food waste behaviour: a systematic review | Appetite, 176 | 2022 | Blue | 0 | 0 |
| Alazaiza M.Y.D. (2022) | Alazaiza M.Y.D.; Abdelfattah F.A.M.; Almaskari T.; Bashir M.J.K.; Nassani D.E.; Albahnasawi A.; Abushammala M.F.M.; Hamad R.J. | Effect of Covid-19 pandemic on food purchasing and waste generation during the lockdown period in the sultanate of Oman | Global Nest Journal, 24(1), 59-64 | 2022 | Blue | 0 | 0 |
| Muresan I.C. (2022) | Muresan I.C.; Harun R.; Andreica I.; Chiciudean G.O.; Kovacs E.; Oroian C.F.; Brata A.M.; Dumitras D.E. | Household attitudes and behavior towards the food waste generation before and during the Covid-19 pandemic in Romania | Agronomy, 12(3) | 2022 | Blue | 0 | 0 |
| Bench M.L. (2005) | Bench M.L.; Woodard R.; Harder M.K.; Stantzos N. | Waste minimisation: home digestion trials of biodegradable waste | Resources, Conservation and Recycling, 45(1), 84-94 | 2005 | Yellow | 25 | 1 |
| Diaz-Ruiz R. (2018) | Diaz-Ruiz R.; Costa-Font M.; Gil J.M. | Moving ahead from food-related behaviours: an alternative approach to understand household food waste generation | Journal of Cleaner Production, 172, 1140-1151 | 2018 | Yellow | 72 | 0,8257 |
| Attiq S. (2021) | Attiq S.; Chau K.Y.; Bashir S.; Habib M.D.; Azam R.I.; Wong W.-K. | Sustainability of household food waste reduction: a fresh insight on youth’s emotional and cognitive behaviors | International Journal of Environmental Research and Public Health, 18(13) | 2021 | Yellow | 5 | 0,5912 |
| Allison A.L. (2022) | Allison A.L.; Lorencatto F.; Michie S.; Miodownik M. | Barriers and enablers to food waste recycling: a mixed methods study amongst UK citizens | International Journal of Environmental Research and Public Health, 19(5) | 2022 | Yellow | 1 | 0,2277 |
| Xu W. (2016) | Xu W.; Zhou C.; Cao A.; Luo M. | Understanding the mechanism of food waste management by using stakeholder analysis and social network model: an industrial ecology perspective | Ecological Modelling, 337, 63-72 | 2016 | Yellow | 20 | 0,2151 |
| Bishop M. (2019) | Bishop M.; Megicks P. | Waste not, want not!: qualitative insights into consumer food waste behaviour | Wit Transactions on Ecology and the Environment, 231, 297-308 | 2019 | Yellow | 2 | 0,0755 |
| Pan Y. (2022) | Pan Y.; Li M.; Guo H.; Li Y.; Han J. | Influencing factors and reduction of domestic solid waste at University Dormitory in Shanghai, China | Scientific Reports, 12(1) | 2022 | Yellow | 0 | 0 |
| Mejia D. (2021) | Mejia D.; Diaz M.; Charry A.; Enciso K.; Ramírez O.; Burkart S. | “Stay at home”: the effects of the Covid-19 lockdown on household food waste in Colombia | Frontiers in Psychology, 12 | 2021 | Yellow | 0 | 0 |
